# Animal Model Alternatives in Filovirus and Bornavirus Research

**DOI:** 10.3390/v15010158

**Published:** 2023-01-04

**Authors:** Lina Widerspick, Johanna Friederike Steffen, Dennis Tappe, César Muñoz-Fontela

**Affiliations:** 1 Bernhard-Nocht-Institute for Tropical Medicine, 20359 Hamburg, Germany; 2 German Center for Infection Research (DZIF), Partner Site Hamburg-Luebeck-Borstel-Riems, 38124 Braunschweig, Germany; 3 National Reference Center for Tropical Pathogens, Bernhard-Nocht-Institute for Tropical Medicine, 20359 Hamburg, Germany

**Keywords:** filoviruses, bornaviruses, microphysiological systems, organoids, organs-on-chips, animal models

## Abstract

The order *Mononegavirales* contains a variety of highly pathogenic viruses that may infect humans, including the families *Filoviridae*, *Bornaviridae*, *Paramyxoviridae*, and *Rhabodoviridae*. Animal models have historically been important to study virus pathogenicity and to develop medical countermeasures. As these have inherent shortcomings, the rise of microphysiological systems and organoids able to recapitulate hallmarks of the diseases caused by these viruses may have enormous potential to add to or partially replace animal modeling in the future. Indeed, microphysiological systems and organoids are already used in the pharmaceutical R&D pipeline because they are prefigured to overcome the translational gap between model systems and clinical studies. Moreover, they may serve to alleviate ethical concerns related to animal research. In this review, we discuss the value of animal model alternatives in human pathogenic filovirus and bornavirus research. The current animal models and their limitations are presented followed by an overview of existing alternatives, such as organoids and microphysiological systems, which might help answering open research questions.

## 1. Filoviruses and Bornaviruses—Two Distinct Families within the Order *Mononegavirales*

The *Mononegavirales* order comprises a variety of single-stranded, negative-sense RNA viruses that encode a non-segmented, linear ORF core set [1,2]. This order contains 11 viral families, including the families of the *Filoviridae*, *Bornaviridae*, *Paramyxoviridae*, *Pneumoviridae*, and *Rhabdoviridae* virus families [3].

Some members of the *Filoviridae* family are notorious for their high pathogenicity in humans. When identified in 1967, Marburg virus (MARV) caused the first known filovirus outbreak [4], followed by the discovery in 1976 of Ebola virus (species *Zaire ebolavirus*, EBOV) in the Democratic Republic of Congo (DRC) and Sudan virus (species *Sudan ebolavirus*, SUDV) in South Sudan (reviewed in [5]). Between 2013 and 2016, an epidemic of EBOV disease of unprecedented magnitude took place in West Africa and caused more than 10,000 deaths [6]. At the time of writing, a recent outbreak of SUDV disease in Uganda with 55 deaths has possibly been subdued [7,8].

It is suggested that most human filovirus outbreaks start by zoonotic spill-over, which may include contact with non-human primates (NHP) and other mammalian species such as duikers, swine, and bats [9]. However, recent research also indicates that, in humans, the virus can persist in immune-privileged sites such as the gonads and the central nervous system (CNS) for long periods of time and that survivors can transmit the virus to naïve individuals, thereby starting new outbreaks [10]. Different species of bats are proposed as potential natural reservoirs in which filoviruses are maintained in nature [9].

Filovirus infections in humans are characterized by high case-fatality ratios (CFRs). For EBOV, Sudan virus (SUDV), and Bundibugyo virus (species *Bundibugyo ebolavirus*, BDBV), the incubation period ranges from 2 days to 21 days with a CFR from 25% to 90% [11,12,13,14]. Reston virus (species *Reston ebolavirus*, RESTV) is presumably apathogenic in humans, although seroconversions have been detected [15]. There is only one known case of human Taï Forest virus (TAFV) infection, which was non-lethal [13,16,17]. Furthermore, the CFR of marburgviruses, MARV and Ravn virus (RAVV), lies between 24% and 88% [18].

During early stages of filoviral hemorraghic fever, patients experience general symptoms of febrile illness, which in severe cases progress towards systemic disease with gastrointestinal symptoms and respiratory and neurological manifestations. Rare hemorrhagic events and more frequent maculopapular or petechial rash are further hallmarks of filoviral disease in humans (reviewed in [5,19,20,21,22,23]).

The primary target cells of filoviruses are probably macrophages and different subsets of dendritic cells (DCs). In vitro, infection of DCs with filoviruses inhibit their capacity to activate T cells (reviewed in [5,19]). Recently, data obtained in clinical studies and animal models also indicate that the DC-T cell crosstalk is an important checkpoint in human filovirus infection [24,25]. Migratory dendritic cells also likely spread the virus from the sites of infection to the tissue-draining lymph nodes, therefore contributing to virus dissemination [24]. The high levels of inflammation associated with severe filovirus infection also dampen the transition between innate and adaptive immune responses. In severe cases, bystander T cell activation, low clonality, and an exhaustion-like phenotype are associated with poor viral clearance and fatal outcome [26,27,28] and are a hallmark of filoviral disease. Fatal filoviral hemorrhagic fever is further associated with massive fluid loss, leading to hypovolemic shock as well as multi-organ failure with coagulopathy, convulsions, and substantial metabolic disturbances. Alternatively, survivors recover with a prolonged convalescent phase and sometimes sequelae such as hepatitis, uveitis, paralysis, hearing impairment, and mental post-traumatic disease (post-ebola syndrome) (reviewed in [5,19,20,21,22,23]).

The zoonotic potential and human pathogenicity of some members of the *Bornaviridae* family has only been shown recently in severe and eventually fatal encephalitis cases. Bornavirus encephalitis has so far been reported following infection with the Borna disease virus 1 (BoDV-1) or the related variegated squirrel bornavirus 1 (VSBV-1) [29,30,31]. In humans, both zoonotic bornaviruses cause an encephalitis syndrome consisting of headache, malaise, fever, and confusion, followed by a multitude of neurological symptoms such as epileptic seizures, hallucinations, muscle spasms, ataxia, and tetraparesis. The symptoms accumulate over several weeks, leading to coma and death of the patient after a few weeks [30,31,32,33,34,35,36].

For a long time, BoDV-1 was only known to cause CNS infection in animal dead-end hosts such as horses, donkeys, rabbits, sheep, cattle, goats, or dogs, as no natural reservoir was known (reviewed in [37]). Here, the infection is limited to the CNS [38]. In contrast, in recently discovered natural reservoir hosts, such as the bicolored white-toothed shrew (*Crocidura leucodon*) for BoDV-1 and exotic squirrels (*Sciurinae* and *Callosciurinae* families) for VSBV-1, systemic infections are seen despite no signs of clinical disease, and virus is shed by infected animals in saliva, urine, feaces, lacrimal fluid, and skin swabs [38,39,40,41]. In infected cells, bornaviruses show persistence intranuclearly [42] and thus establish a chronic infection.

In contrast to VSBV-1 with only five confirmed human cases so far [31,43,44], the emerging BoDV-1 is increasingly reported with more than 40 confirmed and fatal cases in Germany [34]. Most of these cases were diagnosed in retrospect since the first confirmed human cases in 2018 [29,30]. Despite the low case numbers, the combination of a very high CFR [36,45] with a rapid course of the disease [29,33] makes bornaviruses a virus family of increasing importance for public health.

## 2. Filovirus Animal Models

**Figure 1:**
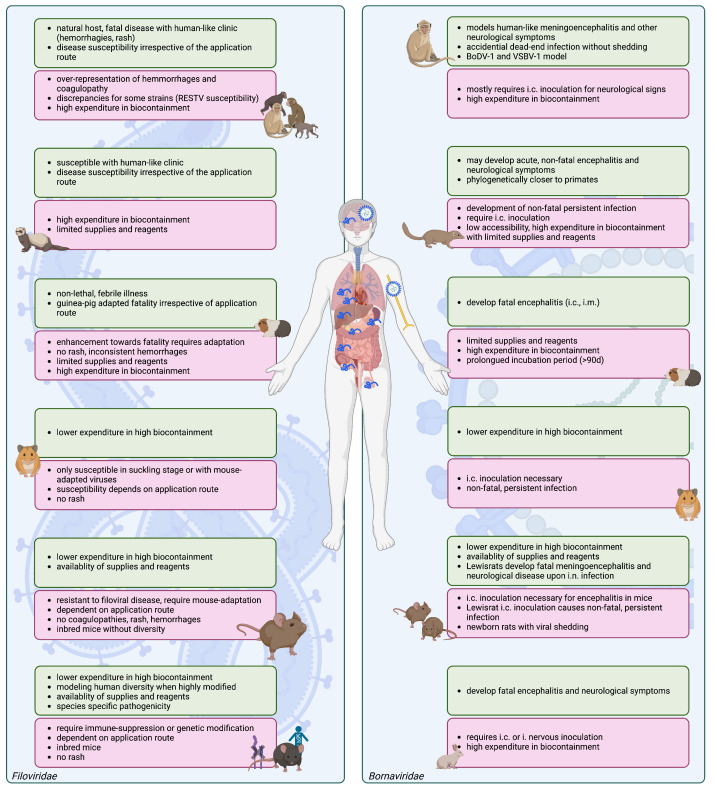
Capabilities and limitations of established animal models in filovirus and bornavirus disease. Scheme of human-pathogenic *Filoviridae* and *Bornaviridae* animal models. Left: Filovirus animal models: non-human primates, ferrets, guinea pigs, hamsters, wild-type mice, and immune-deficient or humanized mice. Right: common animal models for human bornavirus encephalitis including non-human primates, shrews, guinea pigs, hamsters, mice, rats, and rabbits. Expenditure summarizes cost, availability, personnel and space requirements. Created with Biorender.com.

Animal models in filovirus research have already been extensively reviewed elsewhere [19,20,21,22,46,47,48] (Figure 1). They are widely used to understand the viral pathogenesis and immune response and have been crucial for the development of vaccines and therapeutics (reviewed in [20]). Still, many of these medical countermeasures turned out not to be effective in humans, underscoring the translational gap between pre-clinical and clinical studies in filoviral disease.

After 50 years, NHP models, especially cynomolgus and rhesus macaques, are still considered the gold standard models for filovirus research, as they are naturally susceptible to filoviral infections. In NHP, filoviral disease, caused by EBOV, is uniformly fatal [16,49,50,51,52,53,54,55,56,57,58,59,60,61,62]. While early disease signs in NHPs resemble those in humans, in later stages of disease, NHPs more often than in humans progress towards visceral hemorrhages [49,52,59,63,64,65] and coagulopathy [51,57,63,66]. A possible limitation of NHP studies is their applicability to test the pathogenicity of newly discovered filoviruses for humans, due to the fact that RESTV can still cause severe disease in NHP [67,68].

Similarly, ferrets are susceptible to wild-type filoviral infection, succumbing to the disease with human-like symptoms including rash, coagulopathy, hemorrhages, and multi-organ failure [11,14,69,70,71]. In this model, filoviruses have a tissue tropism and disease hallmarks comparable to those of humans [11,14].

In guinea pigs, filoviruses naturally only cause a non-lethal febrile illness [49,56,72,73,74], which can be enhanced towards fast lethality via animal-to-animal passages (guinea pig-adapted (GPA)-viruses) [69,74,75,76]. Interestingly, this is not dependent on the infection route [75]. Like humans and NHPs, macrophages and dendritic cells are primary targets and contribute to the systemic spread of GPA-viruses [69,73,75,77]. Both ferrets and guinea pig models are limited by the availability of reagents and standardized assays.

Wild-type infection with EBOV and MARV does not cause disease in adult Syrian Golden hamsters [70,78,79], whereas suckling hamsters are susceptible [78]. In contrast, mouse-adapted (MA)- or hamster-adapted (HA)-EBOV infection of six weeks old hamsters is fatal within four to five days, depending on the infection route [20,70,79]. Hamsters develop fever, coagulopathies, and hemorrhages, modeling human-like late-stage symptoms [70,79]. Neurological symptoms may develop upon intracranial (i.c.) injection (reviewed in [20]).

Like hamsters, inbred, adult laboratory mice are resistant to filoviral disease [80,81,82]. To achieve susceptibility to diseases in adult mice, many have generated mouse-adapted MA-MARV and MA-EBOV by serial passaging in suckling mice [83] or severe combined immunodeficiency (SCID) mice [82,84,85]. SCID mouse infection with wild-type filoviruses is mostly lethal within 20 to 30 days [19,80,81,82,83], although persistent infection of 50 days to 70 days may occur [86]. Higher and faster lethality arises upon passaging of the virus [82,85]. Wild-type, adult mice infected with such MA filoviruses uniformly succumb within five to eight days without displaying hemorrhages or rash [57,80,83,85,87]. One advantage of the model is that the target cells and tissue tropism are comparable to humans [83,84,85,87,88]. For mice, the route of application determines severity [80,83,84,85,87].

More recently, advanced mouse models susceptible to wild-type filoviruses have been developed. IFN-α/β receptor (IFNAR) or signal transducer and activator of transcription 1 (STAT1) knock out (KO) mice [13,80,89,90,91] have an intact innate, humoral, and cellular adaptive immunity but lack the type I interferon response, which is the main antiviral cytokine and plays an important role in bridging innate and adaptive immune responses (reviewed in [92]). Upon infection, they lose weight and show systemic viral replication when succumbing [13,47,80]. In the IFNAR^*KO*^ mouse model, the interferon competence of the hematopoeitic compartment can be restored by generating bone marrow chimeras using bone marrow progenitor cells from immune-competent donor mice [24]. This way, different levels of pathogenicity of filoviral species and human-like tissue tropism can be modeled [24], overcoming limitations of the full knock out model. More recently, humanized mouse models or even avatar mice harboring DCs and T cells from individual human donors have been generated [25,93].

Humanized mice express human genes or contain human cells or tissues [94]. The immune system of immune-compromised mice such as NOD–SCID–IL-2γ receptor^*KO*^ (NSG-A2) or SCID mice can be reconstituted via human hematopoeitic stem cells, potentially also with primed immune cells or fetal tissues [25,93]. Here, depending on the donor [25] and engraftment [95], human tissue tropism and virus species-specific pathogenesis may be observed upon wild-type filoviral challenge in the context of a particular human donor [95,96]. One advantage of such avatar and humanized mouse models is that they reproduce several important features of the human disease, such as virus dissemination, liver failure, and high levels of inflammation [25,96]. Similarly, humanized collaborative cross-resource recombinant inbred inter-crossed mice (CC-RIX) display broader diversity in their response to EBOV challenge and show a range of disease severity, which may include coagulopathies [97].

## 3. Animal Models in Bornavirus Research

Animal models in bornavirus research have been reviewed elsewhere [38,98]. Prior to the confirmation of BoDV-1 as the pathogen causing encephalitis in humans in 2018, research until the early 2000s suggested a possible link between BoDV-1 infection and various psychiatric conditions [99,100,101]. Studies with animal models have therefore often focused on investigating possible psychiatric aspects. However, high variability in psychiatric patient groups and high seroprevalence in healthy control groups has led to controversies in the field of BoDV-1 about the putative role in human psychiatric diseases. After that, a large study evaluating samples of 396 subjects by multiple assays strongly argued against a role of bornavirus in psychiatric disorders [102].

Only recently were BoDV-1 and the newly discovered VSBV-1 shown to induce a meningoencephalitis and other neurological symptoms in rhesus macaques, closely resembling human disease [103,104]. Intracranially (i.c.), BoDV-1- or VSBV-1-infected animals initially displayed general symptoms of anorexia, sleepiness, and decreased activity, and they developed severe neurological signs, including focal myoclonus, tremors, and behavioral changes [103]. Interestingly, VSBV-1 neurological symptoms upon i.c. inoculation were less severe, rather showing behavioral changes [103]. Similarly to human cases, lymphocytic perivascular cuffing and meningeal inflammatory infiltrates were detected in i.c.-infected NHPs. Like in humans, VSBV-1-infected NHP post mortem analyses revealed a strict central neurotropism, while for BoDV-1 RNA also appeared in peripheral nervous tissue in few other organs [103].

Strict neurotropism is also a hallmark in the most often used BoDV-1 animal model, the inbred Lewis rat. Rats may develop fatal meningoencephalitis and neurological disease upon i.n. infection or infection achieved by co-habituating infected, asymptomatic newborns with mother rats [105,106,107,108]. In this way, BoDV-1 intra-axonally migrates from the neuroreceptors within the olfactory epithelium into the brain, establishing fatal disease with strict neurotropism and without shedding [105,109]. Here, first olfactory nerves, then later parts of the olfactory system, and finally di- and telencephalon, including hypothalamus, thalamus, cortex, and hippocampus, as well as late presence in the cerebellum and medulla oblongata, were detected, and 90% of the rats died after one week of symptom onset, within a month after infection [105]. In contrast, i.c. inoculation of rats with BoDV-1 causes non-fatal, acute meningoencephalitis and subsequent persistent infection associated with chronic debility and loss of brain tissue [105,106,107,108,109], prominently differing from the NHP model [103]. In Lewis rats, it is suggested that BoDV-1 meningoencephalitis and brain lesions are associated with immunopathological effects of the CD4+ and CD8+ T cell subsets [110,111,112,113].

Importantly, the incubation period and persistence of BoDV-1 or VSBV-1 in humans are unknown, while the time until death after symptom onset is short (weeks to a few months). However, a few chronic human cases exist, likely associated with iatrogenic immunosuppression [36]. As a result, both models, one highlighting persistent infection and the other focusing on acute disease, might be suitable to represent human disease in terms of different aspects. Newborn rats are persistently infected but do not show any signs of disease. Lewis rat neonatals further do not have a strict neurotropism and shedding of BoDV-1 [105]. As a result, neonatally infected rats do not resemble the human host but rather the reservoir species [38,39].

Adult laboratory mouse models were described as mostly persistent but asymptomatically infected [114,115], while hamsters, when i.c. infected, develop non-fatal persistent infection [116], which is also seen in Lewis rats. Upon serial mouse passaging of BoDV-1, mice may, however, progress towards fatal encephalitis upon i.c. infection [114,115,117,118], which is possibly associated with an immunopathological CD8+ T cell response [118]. In more historic models generally used to generate viral stocks from tissue homogenates, guinea pigs also develop fatal encephalitis upon i.c. and i.m. inoculation with a comparably long incubation period of a minimum of 90 days [119]. Similarly, in rabbits, fatal encephalitis was observed upon i.c. or intranervous inoculation three weeks until eight months after infection as well [116,120,121]. These models were, however, never developed further as *bornaviridae* animal models.

## 4. Complex In Vitro Models in Viral Research

### 4.1. 2D In Vitro Models

Naturally, 2D modeling is used to study singular, specific aspects of disease in a maximally controlled system. In molecular virology and basic research, such systems can therefore be the most straightforward approach. In more translational research, virus immunology, and pathogenesis studies, including candidate drug screening and toxicity testing, however, mostly models are used. Here, 2D models are predecessor systems that complement animal models, providing a platform with less complexity and increased throughput capacity [122].

While 2D cell cultures have been successfully applied for antiviral screening, it is often the case that the therapeutic potential of these drugs does not translate well to animal models. Well-established and widely used culturing of mammalian cells over a long period of time often comes with a loss of tissue-specific architecture and function, innate immune competency, or changes in the differentiation state of the cells [123,124,125,126]. Some of these drawbacks can be overcome by the use of primary cell cultures and explants from donors, as well as donor stem-cell-derived cells [126]. Indeed, primary human brain-derived neural stem/progenitor cells attained from fetal tissue have been used to study BoDV-1 infection, including the effect on neurogenesis and astrogliogenesis upon differentiation [127,128]. However, donor-to-donor variations, limited accessibility, and short lifespan substantially limit primary cell practicability [126,129]. Nonetheless, induced pluripotent stem cell (iPSC)-derived human cells such as hepatocytes are physiologically more similar to human primary hepatocytes than the well-established Huh7 cell line, thereby better modeling the host cell response, for instance, in filoviral infection [130]. Moreover, primary cell culture may be further advanced by micropatterned co-culture (MPCC) [129,130,131] to model a certain niche with adequate cell–cell-interactions. Such models can be more predictive of clinical outcomes compared to standard culture [129,131]. As such, co-culturing of immune cells to elucidate innate and adaptive immune responses is a common tool in filovirus and bornavirus research [108,132,133,134].

### 4.2. Organoids and Other Static 3D Cell Culture Models in Viral Research

One major drawback of 2D cell culture is the lack of complex tissue function, microenvironment, differentiation, and tissue organization. Consequently, 3D cell culture models were developed with the hope of approximating the spatial and chemical complexity found within a true organ. In 3D cell-culture models, tissues are either microengineered, printed, or grown in extracellular matrix (ECM) to acquire the cellular polarization found [123,124].

Spheroids inherently aggregate into 3D tissue clusters made up of at least one immortal or primary cell type [135]. They are often mislabeled as organoids, although they lack key features of organoids such as self-organization, generation from stem cells, and adequate tissue architecture [135]. They do, however, have the potential to display cell–cell interactions in a more relevant 3D context than conventional 2D cell systems.

In contrast, organoids are self-organized 3D tissue clusters that are driven from human embryonic stem cells (hESC), adult stem cells (AdSC), or iPSCs [122,136,137,138,139]. They mimic human organogenesis, epigenomic, and transcriptomic signatures and may even contain immune-competent cells [135,136,139,140,141]. Organoids are sequentially derived in the desired organ from a commercial stem cell line or reprogrammed cells from donors. These organoids have the capacity to semi-autonomously simulate the required developmental cues to form a mature organ structure [139]. Here, organoid three-dimensionality is generally achieved through the aggregation or embedding of cells into a 3D matrix (reviewed in [135,139]). As a result, histogenesis and physiology in particluar, including drug metabolism and cytotoxicity, have been recapitulated (reviewed in [123]).

Generating iPSC from a donor comes with the unique advantage of a limitless supply of different models that can outlive the patient’s lifespan [139]. To generate such iPSC-derived organoids, each tissue requires a unique protocol and growth factor gradients that drive the cells towards their desired fate by (in-)activating key signaling pathways of development. Here, the germ layers, i.e., endoderm, mesoderm, or ectoderm, have to be initially specified (reviewed in [139]). For instance, in the case of brain organoids, preliminary generation of embryoid bodies and subsequent differentiation into the neuroectordermal lineage is achieved via Wnt and BMP4 signaling (reviewed in [139]). Endodermal organoids, including some gastrointestinal organoids or the lungs, only progress via laborious step-wise differentiation protocols in which timing and growth-factor gradients are crucial to achieve the right organ (reviewed in [139,142]).

AdSC organoids are generated from patient biopsies and tissues by embedding stem cells in a 3D matrix and providing the required niche factors to maintain their stemness (reviewed in [139]). As a result, the stem cells produce epithelial monolayers of a certain organ that mimic their architecture [139]. To maintain such AdSC organoids, in theory indefinitely, they are regularly passaged in precisely refined conditions (as reviewed in [139]). Therefore, like iPSC-derived organoids, they can be used to long-term store patient-specific biological information [139].

#### Assembloids

Assembloids provide an additional step of complexity when compared to organoids by combining organoids of different tissues or adding other cell lineages and thereby enabling more complex scientific approaches.

Multi-lineage assembloids are created by introducing primary cells or PSC-derived cells from distinct lineages to the organoid [143]. Accordingly, the combination of microglia-like cells and cortical organoids was previously used to model certain aspects of Alzheimer’s disease [144]. Furthermore, the introduction of pericyte-like cells enabled the successful infection of cortical organoids with SARS-CoV-2 [145]. The introduction of immune cells to organoids is especially highly desired in logical and immunological research. Furthermore, innervation or vascularization by applying neuronal progenitor cells or endothelial cells is being explored to phenocopy human organs [135].

Another approach is the combination of organoids of one lineage that represent different regions into one multi-region assembloid [143], for instance, combining ventral with dorsal forebrain organoids [146]. Alternatively, polarized assembloids show spatial topographic organization that can be induced by a gradient. This way, Sonic Hedgehog (SHH)-secreting cells can generate a gradient that, in forebrain organoids, can polarize the organoids insofar as lateral and medial ganglionic eminence, hypothalamus, thalamus, and dorsal forebrain regions form [147].

Many characteristics of viral disease have already been modeled in spheroids, organoids, and assembloids. This ranges from Zika virus microcephaly to a massive SARS-CoV-2 research output during the 2020s pandemic covering lung, brain, cardiac, choroid plexus, gut, intestinal, and colon systems [138,142,148,149,150,151,152,153,154,155,156,157,158,159,160]. Here, human PSC- or AdSC-derived alveolar and lung organoids, as well as AdSC-derived intestinal and nasal mucosa organoids, were explored in SARS-CoV-2 infection assays to screen for potential (re-purposed) drugs or general investigation of tropism and antiviral response (reviewed in [142]).

Very early application of organoids in viral research included brain organoids modeling whole brain development or individual regions of interest [159,161]. By recapitulating the cytoarchitecture of the fetal brain, these recapitulate human-specific structural phenotypes of viral infection, which may not be modeled in animals [158,159,161]. This way, morphological and transcriptional changes in Zika virus (ZIKV), human cytomegalovirus (HCMV), and herpes simplex virus 1 (HSV-1) have been identified, which is consistent with a microcephaly-like phenotype reported upon in utero infection [158]. Remarkably, such observed cytoarchitectural changes have not been reported in 2D systems, in this case indicating advantages of the organoid model [158].

**Figure 2:**
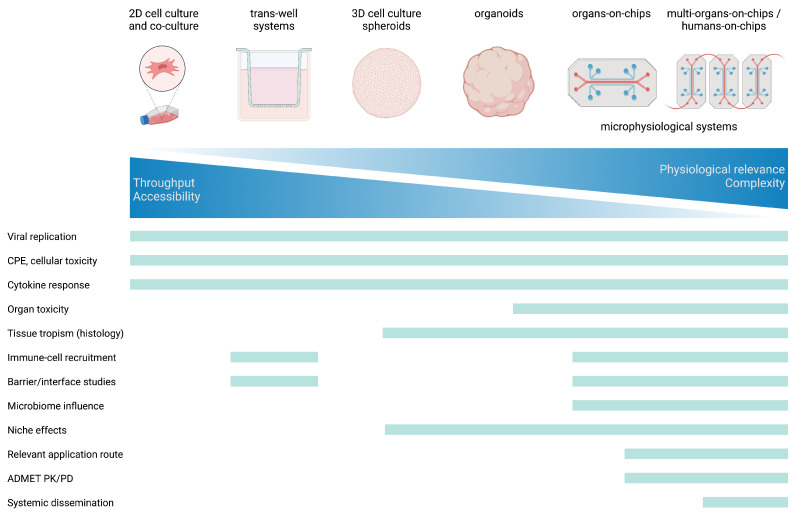
Capabilities and limitations of systems logy. Summary of experimental read-outs attainable in the respective systems. Created with Biorender.com.

When studying such 3D models of infections, imaging and sequencing, as well as measuring viral production, are common experimental parameters (Figure 2). Thereby, viral replication kinetics and cytopathic effects (CPE) are observed in a tissue-specific and microenvironment-dependent manner, where a certain application route can be investigated. Furthermore, metabolic output can be captured via the multiplexed measuring of cytokines as well as quantitative mass spectrometry of the samples. Alternatively, organoids from different species such as bats can be used to elucidate host or reservoir specific patterns, as well as production of viral stock of viruses that are hard to propagate conventionally [150].

The increasingly available single-cell RNA sequencing and proteomics as well as spatial transcriptomics platforms further leverage the full potential of organoids, where cytoarchitectural read-out and single-cell resolution impressively capture viral pathogenesis. As a result, (donor-dependent) cell composition and transcriptional signatures were detected in bronchioalveolar organoids infected with influenza virus or distal lung organoids infected with SARS-CoV-2, among others [152,162]. Additionally, advances in including immunocompetent cells [141], generating assembloids, and microbial co-infection [163] in these models are increasingly being applied. Indeed, Purwada et al. engineered mouse immune 3D culture recapitulating the B-cell zone of lymphoid tissue, thereby generating B cells with a germinal center-like phenotype comparable with naive murine B cells [164].

To conclude, organoids contain primary human cells in a physiologically relevant microenvironment, which, in viral research, provides a stage to reproduce autonomous viral effects (e.g., CPE) and non-autonomous effects of cytokine and metabolite stimuli of an infection environment in nearly real time [136]. Moreover, as many organoids have a polarization into a basal and apical site, different application routes may be studied [152].

### 4.3. Organs-on-Chips as Microphysiological Systems under Constant Fluid Flow in Viral Research

The first approaches towards using microphysiological systems (MPS), especially of barrier function, were undertaken in trans-well cultures. Here, cell culture inserts were used to generate multiple reservoirs within a well, separated by a porous membrane. This way, simple barrier structures such as the endothelial layer or air–liquid interface (ALI) can be reconstructed [142]. Once fluid flow is applied to microfluidic channels, such systems can be transformed into organs on chips (OOCs), which are commonly defined as microengineered micofluidic cell-culture devices. They contain integrated tissue-specific cells that are continuously nurtured by flowing through a microfluidic channel system [123,124,165]. When under flow, such an OOC supposedly mimics a desired tissue microphysiology, including its microarchitecture, tissue–tissue interfaces, cell composition, polarity and position, vascularization, mechanical cues (shear force, torsion, stress, tension), and extracellular matrix (ECM) with the respective chemical gradients [124,165,166,167]. In brief, OOCs are microphysiological systems that make up minimal functional units of the tissue they aim to recapitulate [124]. In their most basic function, they are made up of hollow channels lined by cells (i.e., endothelium) and other tissue-specific cells that are consequently under continuous fluid flow [123]. In their most developed form, OOCs are highly evolved microengineered devices, which include patient-specific organoids or cells, providing personalized pre-clinical models with tissue microphysiology and -pathology [123,168].

In general, OOCs are not a novel idea and were already microfabricated in the 1990s via photo-/soft-lithography. This technique uses high-intensity ultraviolet (UV) light to etch microstructures into photosensitive layers. These can finally be used as casts for replica molding [169]. Consecutively, molding of poly(dimethylsiloxane) (PDMS) onto the etched structures produces a complementary, optically translucent rubber recapitulating the desired channel and chamber system when covered with a plane of any desired material [169,170,171,172]. More recently, manufacturing techniques such as 3D printing, laser etching, or injection molding, among others (reviewed in [124]) have been additionally applied. When using 3D printing and microengineering, microstructures can be printed as tubular systems, which are continuously perfused and subsequently colonized by spatially defined cell types in region-specific ECM gels [123].

When unperturbed, flow in microfluidic channels of less than 1 mm diameter is entirely laminar [124]. This can, for instance, be leveraged for creating compound gradients [173] used in cell motility and chemotaxis studies [174,175], tissue formation, differentiation, toxin response, and cell–cell junction integrity (reviewed in [124]). It is noteworthy that tissue shear stress may further be adapted via the channel diameter and flow rates, insertion of nanoporous membranes in between the flow and cellular compartments, or physical barriers, e.g., posts, that prevent cells from passing through certain areas (reviewed in [124]). In fact, pumps creating pulsed flow patterns of hemodynamic stress [176], suction for cyclic mechanical strain of a lateral wall and the attached membrane, electrical fields to pace contractile units [177] and stimulate wound healing, or compression may be applied (as reviewed in [124]). When combining a multitude of mechanical stress factors, the physiological movement of organs may be copied.

The integration of porous layers in between microchannels or cell types further provides a platform to analyze processes of tissue–tissue interfaces, such as tissue barrier function, transcellular transport, and absorption or secretion analysis (reviewed in [124]). This is highly important for barrier analyses (e.g., blood–brain-barrier (BBB)) or to study the interface of endothelium and organ parenchymal tissue [124]. In fact, the addition of circulating immune cells into the flow, as well as resident immune cells into local ECM gels, may even allow tracking their function in a local environment (as reviewed in [123,165]).

Immune-competency via the addition of peripheral blood monocytic cells (PBMCs) or specific immune cell types is increasingly common in OOCs [178,179]. As a result, recent advances in lung and intestine OOCs allow for safety profile evaluation of immunotherapies in tumor treatment [179]. Here, cytokine release, change in transcripts, flow cytometry, immunofluorescence microscopy, and the attachment of immune cells may be employed as read outs to gather toxicity and safety data [179].

As they are microfabricated, OOCs are modular with ranging complexity levels. This way, electrical, chemical, mechanical, and optical probes used for the direct read-out of the organ-on-chip may be integrated within the microdevice [180,181]. The micropattern in which cells are contained within an OOC determines the reconstruction of a tissue’s functionality. Cells may either be plated into the chips via the laminar stream within the microchannel, selectively adhering to specific ECM molecules previously coated [170,171,172,182], or cells may further be positioned by using microbarriers to determine the areas in which they can venture [183]. They can also be directly 3D-printed into the desired area [123]. In theory, there are no limitations of cell types or species, given that appropriate culturing conditions can be met [184]. However, fulfilling the needs of all cell types requires immensely complicated co-culturing.

Due to their limited lifetime of approximately a month, organs-on-chips are more likely used in research for acute diseases [124,185]. Organs, or rather tissue units modeled by chips, so include, but are not limited to, the lung, CNS (brain, BBB), blood vessels, cartilage, eye, fat, heart, immune system, muscle, small intestine, colon, kidney tubule, kidney glomerulus, liver, lung alveolus, lung airway, mammary gland, nerve, pancreas, placenta, skin, teeth, and uterus (reviewed in [123]). Moreover, there are bone-marrows-on-chips which recapitulate hematopoeisis via the co-culture of human hematopoietic stem cells and bone marrow stromal cells in a microfluidic device [186].

#### Multi-Organ-Systems- and Humans-on-a-Chip

In pharmacological research, modeling absorption, distribution, metabolism, excretion, and toxicity (ADMET), pharmacokinetics (PK)/ pharmacodynamics (PD) is one of the major goals of MPS modeling. By identifying PK and PD parameters, together with information on ADMET, an efficacious human dose, as well as the safety and toxicity profile of a drug, may be determined [187]. However, many (human) organs or tissues therefore have only been modeled with OOCs as isolated entity. This, however, fails to represent the diverse functional units of the body and especially cannot model drug–body interactions.

To overcome this, physiologically aligned 2D cell cultures, or so-called micro-cell culture analog (microCCA) systems, representing different compartments have been utilized to model ADMET in drug testing [188,189,190,191]. In this way, Miller and colleagues interconnected 13-organ cell lines mimicking the organs and physiological barriers to determine inter-organ drug distribution [192]. Advanced multi-organ-chips- (MOCs) or bodies-/humans-on-a-chip (BOCs) represent microengineered models that reconstitute the human body by functionally integrating multiple chips of organs of the human body or a systematic unit into one fabrication [165,185,193,194]. This means that single OOCs are fluidically coupled to a physiological multi-organ unit to model tissue–tissue crosstalk [123,184,195,196,197]. Such fluidical coupling itself leads to the phenotypic modulation of the cells as well as metabolic coupling of the compartments [196], potentially moving further towards physiological conditions. Indeed, effects already found were reproduced in this system [196]. As disease initiation and progression involves many compartments of a body, shared medium flow where tissue–tissue communication is enabled without hampering the individual tissue identity is required [197]. As a result, different metabolic and nutritional requirements must be met [197].

Accordingly, absorption properties of a 3D intestine and skin biopsy barriers were coupled with metabolizing liver spheroids and a kidney barrier made up of proximal tubule epithelium [185]. Here, peristaltic surrogate blood flow and a second drainage channel for fluid excretion through the kidney epithelium comprise the ADME model [185]. Other MOC are capable in comparing systemic and topical applications of drugs as well as acute and chronic drug exposures [194]. Herland et al. furthermore developed a gut-liver-kidney MOC to determine PK/PD parameters of intravenously administered drugs with an arterio-venous fluid mixing reservoir mimicking systemic circulation [198]. Their endothelial lining of the channels allows for systemic flow of a blood substitute which can be quantified for blood and plasma concentrations of applied drugs [198]. Semi-permeable endothelial separation of compartments further ensures that the connected parenchymal compartments remain specific to their desired target tissue, especially when stem cell-derived lineages are used [197]. A shared vascular channel generally provides an environment for a functional human immune MOC with circulating immune cells such as monocytes, making it a platform that can be used to analyze tissue-specific immune-responses to drugs and damage [197,199,200].

The application of MPS in infectious disease has become more popular. Indeed, the modeling of respiratory infections in lung and airway OOCs [201,202,203,204], hepatitis B virus (HBV) infections in liver chips [142,205], neurotropic viruses in neurovascular units [184,206], and viral infection of the intestine [207,208,209] and kidney [210] are increasingly being explored.

While lung chips have been widely applied in SARS-CoV-2 research, Huh and colleagues already pioneered this work in 2015 by creating a human breathing lung-on-chip model that mimicked the alveolar–capillary interface, hemodynamics, and breathing movements via cyclic suction [167]. The inclusion of mechanical breathing motions in lung chips can substantially influence viral replication and innate immune responses [203], generating a more precise model of the viral environment found. This may cause tissue tropism and phenotypical alteration, including barrier function and regeneration capacity upon viral infection [203]. In a simple two-channel system under constant flow, human alveolar chips can reproduce the alveolar–capillary barrier with an air–liquid interface (ALI) via the co-culture of alveolar epithelial cells, microvascular endothelium, and even circulating immune cells [202]. Accordingly, when infecting the upper alveolar channel with SARS-CoV-2, an adequate infection route may be modeled, while at the same time, PBMC in a lower, vascular channel renders the OOC immune-competent [202]. Reportedly, read-outs such as barrier function, viral tissue tropism and CPE, immune cell recruitment, viral replication, and immune-/inflammatory response, antiviral testing, as well as in situ imaging, even with increased throughput in a 96-well format, are possible [202,204,211]. Accordingly, neurotropic viruses studied in a neurovascular-unit-on-a-chip models were investigated for their BBB crossing, barrier disruption, and neurotropism by live cell imaging, high-performance liquid chromatography, or quantitative mass spectrometry measuring components on either side of a barrier [184], while Villenave et al. designed a multi-channel gut-on-a-chip to query similar parameters in a villus intestinal epithelium under constant flow and peristalsis-like motion [207]. Here, the authors tested different application routes, including direct apical application in the epithelium-lined lumen and introduction via the basal parallel-flowing vascular channel, where they found differences in viral titers and CPE, as well as a preference for apical viral particle release [207]. Intestinal SARS-CoV-2 infection was studied in OOCs as well, including inhibitor screening and toxicity evaluation [208] and immune cell recruitment [209]. Meanwhile, liver chips were employed by Ortega-Prieto and colleagues to investigate all stages of the hepatitis B virus (HBV) replication cycle in a model that displays hepatic sinusoid microarchitecture, bile canaliculi, and cell polarization [205]. Indeed, their liver chip continuously secretes albumin and is metabolically active, providing read-outs commonly acquired in animal modeling [205]. Overall, the authors showed large differences between 2D and 3D static cultures, as well as chips under flow [205].

In filovirus and bornavirus research, the use of organoids and MPS is still at an early stage. Junaid et al. have recently modeled Ebola hemorrhagic shock syndrome on a very simple microvessel chip [212]. In this chip, human umbilical vein endothelial cells (HUVECs) were seeded into microvascular channels in the commercially available MIMETAS OrganoPlate (T-design) [212]. Here, the microvessel channel comprises an inlet and outlet for the medium as well as an observation window suitable for imaging [212]. A second ECM channel with direct contact to the microvessel channel comprises a T-junction, where the diffusion of fluorescently labeled substances, in this case albumin, into the ECM may be employed to determine the influence of applied drugs or viral particles on vascular leakage and integrity [212]. Bidirectional fluid flow through the chip was achieved by placing the device on a rocker [212]. In their experiment, the authors luminally applied Ebola-virus-like-particles and glycoprotein, meaning via the inlet for the medium flowing through the chip [212]. Thereby, cytoskeleton remodeling and subsequent vascular integrity loss during ebola hemorrhagic shock syndrome, which was in fact rescuable via FX06 and melatonin treatment, were imitated [212]. Nevertheless, the chip still lacks many features of an advanced OOC, including co-culture of relevant cell types, uni-directional flow governed by a pump, and addition of immune cells [212].

## 5. Caveats for the Usage of Organoids and MPS in Virological Research

It cannot be overlooked that the use of organoids is a very young technique that is still under constant development. In particular, the increasing complexity when joining multiple components in assembloids renders the system even more vulnerable to heterogeneity and increases culture conditions to be met. In organoids, sometimes spontaneous differentiation of progenitor cells results in the substantial heterogeneity of composition and properties [161]. Thus, even small changes in culture may amount to considerable differences among samples and batches, especially when considering the long-term culturing [124,139,161]. Coherently, good cell culture practice (GCCP) and standardization, especially when scaling the ratios of the components to be joined, is crucial. Furthermore, while organoids represent many more aspects of tissue architecture than 2D models, the microenvironment is possibly still lacking in comparison to the organ they aim to model [139]. They do not suffice in terms of physiologically relevant cellular organization or organ-supportive tissue [213]. Indeed, MPSs, including OOCs and MOCs, with their mechanical cues and gradients, are more accurate in some regards. Particularly, in the case of filoviral disease, where a broad tissue tropism and viral dissemination requires the integration of multiple tissue models. Advanced MPSs include tissue-tissue interfaces, mechanical cues, interstitial flow, circulating immune cells, or vascularization with flow, adequate shear force, spatiotemporal gradients, or torsion [122,165]. Consequently, they may be more optimal in modeling viral kinetics and pharmacological aspects of ADMET and PK/PD of a candidate drug not possible in human patients [123,197]. The compatibility of MPS and organoids with innovative real-time imaging allows monitoring if respective recombinant surrogate viruses are available such as the recombinant EBOV-GFP. Especially for BoDV-1 and VSBV-1, where virus shedding is limited, this would enable monitoring of viral infectious particle kinetics without requiring tissue homogenization. However, no such tools are available for bornaviruses at the time of writing; therefore, organoids and MPS cannot reach their full potential in this case. Additionally, the use of MPS and organoids in high biocontainment requires the facilities to be upgraded according to their operational needs and real-time analyses. This includes making sure that microfluidic devices are safe to operate and decontaminate when used in pathogen research in high-risk groups.

To replace preclinical animal models with alternatives, they have to be rigorously validated and show equivalent or superior performance [123]. Here, such alternatives must provide human-relevant studies that are reproducible, reliable, consistent, and statistically robust irrespective of the laboratory performing them [123]. Such standards are unquestionable despite the well-known fact that even preclinical animal models sometimes fail to meet them. Nonetheless, such a validation process will most likely require the commercialization and definition of device parameters and read-outs depending on a specific context of use rather than a one-size-fits-all validation [123]. This means that a specific device may most likely only be used for toxicity, PK/PD, efficacy, safety, or ADMET in the area of a specific physiological context [123].

At the same time, MPS microfabrication requires down-scaling of physiological parameters to chips [197]. The accurate adaptation of channel size, compartment distance, matrix, and medium formulation crucially determine tissue integrity, differentiation, and function. Moreover, the adaptation of cell type ratios, oxygenation, flow rate, total volume, physiological range of factors, mechanics, residence times in organs, recirculation properties, diffusion parameters, shear stress, and many more must be considered [139,185,195,197,214,215,215] (considerations for MPS design reviewed in [215]). These are usually not in the scope of a non-tissue-engineering laboratory. Consequently, establishing a robust and physiologically adequate model is highly complex and includes validating all parts of an MPS [197,200,215]. Moreover, bubble formation, medium leakage at connections, increased contamination risk, and ECM degradation over time limit the duration that an MPS may be used [197,200].

Finally, the integration of the immune system into organoids and MPS is currently in an early phase, where mostly naïve PBMC or immortal immune cells are added to the culture. As a result, immune memory effects or more advanced immunological phenomena are not yet represented within these systems and require further research.

## 6. Organoids and MPS—The Future of Filovirus and Bornavirus Research?

A substantial proportion of biological processes are evolutionarily conserved in mammals, allowing for great biomedical and translational breakthroughs in research over the last centuries on the basis of animal models. Indeed, NHPs and small mammals have modeled parts of mononegaviral pathophysiology and disease, providing a platform for therapy development and testing in a feasible environment where genetic manipulation is possible [135]. However, it is not rare that findings from animal experiments fail to predict outcomes in humans [123,216,217]. This may partly be due to poor methodology and study design, namely dosing schedules and regimens, animal selection, blinding and randomization, group size, statistical analysis not accounting for laboratory techniques, the selection of parameters, and follow up (review in [216]). Alternatively, translation may simply fail when a certain animal model inherently cannot predict the human response due to its biology [187,197,214,216,218]. Indeed, some biological phenomena simply cannot be reproduced in animal models, for instance, the complexity of the human brain, human-specific organogenesis, human-specific metabolism or anatomy, or (epi-)genetic diversity [135,139,197,219]. This was true for a brincidofovir compassionate use trial by Dunning et al. during the 2014 EVD outbreak, when NHP studies were circumvented on the basis of an altered drug metabolism in the animals [220]. Furthermore, Bailey and colleagues’ work implies that the absence of toxicity in small animal studies does not preclude the absence of adverse drug reactions (ADRs) in humans [217]. At the same time, toxicities in small animals only inconsistently predict ADRs in humans, depending on the compounds and their targets [217]. Accordingly, drugs that are not safe and efficacious in animal models but would have been in humans are discarded too early in pre-clinical trials [123,217].

In filovirus and bornavirus disease animal modeling, possibly all of the above apply at least to some extent. Besides the biological capacity, this gap is also formed because early time-points from filoviral infection until early innate immune response, particularly the role of the type I interferon response, as well as the final stages of disease or persistence, are not sufficiently understood in neither humans nor any animal model [48,57]. Moreover, many human clinical samples, especially biopsies, are principally collected post mortem, and therefore important stages of disease are not represented. Here, the quality and quantity of clinical samples varies substantially. As a result, many findings in animal models cannot be validated with human samples. This is particularly true for bornavirus disease, where only very few, often retrospectively identified, human cases occurred as of today, or filoviral disease, where general outbreak setting and risk of autopsies complicate high-quality sampling. Consequently, human models may address such a knowledge and validation gap.

Inherently, mice fail to reproduce human filoviral disease, unless immunosuppressed, genetically engineered, or humanized. In fact, even given such modifications, their predictive power as preclinical models for therapeutics and clinical disease modeling has limitations [19,57,79,88,123,218] (Figure 1). For instance, in filoviral disease, immunosuppressed mice or inbred mice infected with adapted viruses do not present with coagulation defects, rash or hemorrhages [85,93], while NHPs develop these to an extreme not found in humans [57,80,85,87]. Given the range of symptoms modeled by different animal species, the hemorrhagic presentation of a host might be a consequence of its genetic background and pathophysiology rather than inherent to filoviral disease [48]. As a result, animals, especially when inbred, most likely cannot model the full diversity of a human disease outbreak, ranging from asymptomatic to detrimental [48]. At the same time, it is important to note that pre-existing medical conditions and treatments [57] are not taken into account in most animal models either. Consequently, even if animals reproduced a disease phenotype, the molecular mechanism underlying and consequently the targets proposed might be entirely different than in a particular human case [218]. Here, more laborious avatar models would be necessary [25].

Donor-derived organoids or OOC may therefore be more suitable to represent human diversity and physiology. While the development of adequate animal models requires preliminary information and knowledge about a viral disease, complex 3D systems could be applied faster with less information required, therefore providing a platform to gather knowledge in pathogenesis and potentially immune response that might support subsequent animal model development [139]. In fact, one may generate patient-specific models of individuals showing adverse effects or non-responders in clinical trials to determine factors influencing drug toxicity and efficacy [123,221] or of survivors to determine host factors of disease susceptibility. This may also pave the way towards precision and personalized (patho-)physiological models that may be employed in drug development and choice [123,139,168,221]. Moreover, modeling in nonhuman species further widens the tool-box for host and reservoir screening of emerging diseases [150,219], particularly in the context of zoonoses such as bornavirus disease, where animal models may possibly be closer to a reservoir species, and therefore represent an infection that is not relevant for human disease. Interestingly, next to mouse [222,223] and NHPs [224,225,226], iPSCs already exist for a wide range of domestic species, including pigs [227,228,229], horses [230,231], cattle [232,233], and sheep [234] (reviewed in [219]). Such models would present major advantages, particularly in emerging viruses such as borna- or filoviruses. Having a system in which immediate results in regards to tropism, CPE, or innate immune response may be generated could therefore accelerate the research progress massively. Moreover, advances in immune cell organoids containing T- and B-cells as well as the addition of myeloid cells and vascularization into assembloids might further widen the immunological repertoire of organoids in the future [142,164].

The utility of organoids in outbreak response was observable with the 2020s SARS-CoV-2 outbreak, where researchers did not hesitate to use a multitude of organoid models [150,152,153,154,155,156,157], contributing to the high demand of the pandemic response, including preliminary drug screening [142]. In fact, experiments in organoids may be performed more rapidly, considering the adaptation of viral strains, breeding of disease-adapted animal models, the availability of human-fit reagents, or the permission-granting process required in animal studies. This, together with less involved risk and space requirements when working within high-containment facilities, reduces the expenditure in case of filoviral research, where work is performed in Risk Group 4 facilities.

Infection routes and drug absorption could be investigated in skin, gastrointestinal, lung, or oral mucosa MPSs [197]. Here, such MPSs may bridge a gap already identified in filoviral animal modeling, where different infection routes in mouse models have a massive impact on disease severity. Human systems may consequently be applied to determine the factors influencing such effects. Moreover, in the case of bornaviruses, the infection route in humans can only be assumed. Therefore, relevant MPSs may be crucial to explore all possibilities prior to imposing a route in an animal model. Vascular or lymphatic MPSs with systemic circulation may further generate information on viral dissemination as well as potential drug distribution in a human environment (reviewed in [187,197,198]). More importantly, liver OOCs or contextualized MOCs may be used to explore liver uptake and targeting, (pro-)drug metabolism with human-specific CYP enzyme repertoire, efficacy and liver PD, hepatotoxicity mechanisms, and biliary excretion, whereas kidney MPSs may explore the impact of a disease on the clearance, reabsportion, and nephrotoxicity (reviewed in [187,197,235]. To conclude, MPS may be applied to precisely determine human-tissue-specific parameters of disease not detectable in simple 2D culture and often not detectable in animal models.

For filoviral therapeutics and vaccines, traditional clinical trials with a placebo group are not feasible on the basis of the high virulence and lethality [47]. The FDA Animal Rule thus permits the replacement of human efficacy clinical trials with animal PK, PD, and efficacy studies in preferably more than one species, with the limitation that therapeutics pass phase 1 clinical trials in humans (reviewed in [47]). Hence, not all stages of preclinical trials can reasonably be replaced with alternative models available to date. However, if adequate models were employed, major differences in humans and animals may be identified in early preclinical trials, and appropriate measures can therefor be implemented earlier to prevent the delay or even failure of the clinical development process [131]. Moreover, in the special case of bornavirus infection, where the rapid progress of the disease and the very limited case number strongly affect therapeutical testing, alternative methods could provide the human factor that is missing in animal models.

## 7. Conclusions

It is generally accepted that simple, especially immortal, 2D cell culture models fail to mimic the complexity of an organ or body (system) [122], while more complex cultures tend to be more predictive. Inherently, experiments are usually favored to confirm findings in these models. However, in the *Mononegavirales* families *Filoviridae* and *Bornaviridae*, animal models cannot capture the respective pathogenesis and disease in full for different reasons, highlighting the importance of complex human-derived systems. These can be an addition to or partially replace animal models to bridge the gap between simple models and human biology. In fact, knowledge gained in organoids or MPS may even be applied to generate or improve animal models and find factors that make model organisms permissive to disease. Therefore, compartmentalized, refined animal models in accordance with the research question instead of a one-size-fits-all solution may broaden the filovirus and bornavirus research repertoire [46]. Nevertheless, organoids and MPS quality control and reporting standards are necessary for scientific reproducibility to make them a robust animal model replacement, particularly in pharmaceutical research, as of now [126,214]. Their current capabilities, however, make a compelling case for gradually moving towards complex models in filovirus and bornavirus research.

## Abbreviations

The following abbreviations are used in this manuscript:

ADMET	absorption, distribution, metabolism, excretion, toxicity
AdSC	adult stem cell
ALI	air–liquid interface
BBB	blood–brain barrier
BDBV	Bundibugyo virus
BoDV-1	Borna disease virus 1
CC-RIX	collaborative cross resource recombinant inbred inter-crossed
CFR	case fatality ratio
CPE	cytopathic effect
CNS	central nervous system
DC	dendritic cell
DRC	Democratic Republic of the Congo
EBOV	Ebola virus
ECM	extracellular matrix
GCCP	good cell culture practice
GPA	guinea-pig-adapted
HA	hamster-adapted
HBV	hepatitis B virus
HCMV	human cytomegalo virus
hESC	human embryonic stem cell
HSV-1	herpes simplex virus 1
IFNAR	interferon alpha/beta receptor
iPSC	induced pluripotent stem cell
OOCs	organs-on-chips
MA	mouse-adapted
MARV	Marburg virus
microCCA	micro cell culture analog
MOCs	multi-organs-on-chips
MPCC	micropatterned co-culture
MPS	microphysiological system
NHP	nonhuman primates
PD	pharmacodynamics
PDMS	poly(dimethylsiloxane)
PK	pharmacokinetics
R&D	research and development
RAVV	Ravn virus
RESTV	Reston virus
SCID	severe combined immunodeficiency
STAT1	signal transducer and activator of transcription 1
SUDV	Sudan virus
TAFV	Taï forest virus
TEER	transepithelial/-endothelial electrical resistance
VSBV-1	variegated squirrel bornavirus 1
ZIKV	Zika virus
